# The zinc figure protein ZNF575 impairs colorectal cancer growth via promoting p53 transcription

**DOI:** 10.32604/or.2023.028564

**Published:** 2023-05-24

**Authors:** NING AN, HEQING PENG, MIN HOU, DUOFENG SU, LIU WANG, XIAOGANG SHEN, MING ZHANG

**Affiliations:** 1 Cancer Center, Sichuan Provincial People’s Hospital, University of Electronic Science and Technology of China, Chengdu, China; 2 School of Medicine, University of Electronic Science and Technology of China, Chengdu, China; 3 Department of Oncology, Affiliated Hospital of North Sichuan Medical College, Nanchong, 637000, China; 4 Department of Oncology, Chengdu Pidu District Hospital of traditional Chinese Medicine, Chengdu, China; 5 Department of Gastrointestinal Surgery, Sichuan Provincial People’s Hospital, University of Electronic Science and Technology of China, Chengdu, China

**Keywords:** Colorectal Cancer, ZNF-575, p53, Prognosis

## Abstract

Zinc-finger proteins play different roles in cancer; however, the function of zinc-finger protein ZNF575 in cancer remains unclear. In the present study, we aimed to determine the function and expression of ZNF575 in colorectal cancer. Proliferation assay, colony formation assay, and tumor model in mice were used to investigate the function of ZNF575 after ectopic expression of ZNF575 in colorectal cancer (CRC) cells. RNA sequencing, ChIP, and luciferase assays were used to investigate the mechanism behind ZNF575 regulation of CRC cell growth. The expression of ZNF575 was determined by IHC staining in 150 pairs of malignant CRC tissues, followed by prognosis analysis. We indicated that ectopic expression of ZNF575 inhibited CRC cell proliferation, colony formation and promoted cell apoptosis *in vitro*. Tumor growth in CRC was also impaired by ZNF575 in mice. RNA sequencing, follow-up western blotting, and qPCR results demonstrated the increase of p53, BAK, and PUMA in ZNF575-expressing CRC cells. Further results indicated that ZNF575 directly targeted the p53 promoter and promoted the transcription of p53. Downregulation of ZNF575 was confirmed in malignant tissues, and ZNF575 expression was positively correlated with the prognosis of CRC patients. The present study demonstrated the function, underlying mechanism, expression, and the prognosis-predicting role of ZNF575 in CRC, which indicated that ZNF575 would be a potential prognostic predictor and therapeutic target for CRC and other cancers.

## Introduction

Colorectal cancer (CRC) is associated with high morbidity in both males and females around the world (second in females and third in males), and also has a high mortality rate worldwide (the fourth cause of cancer-related deaths) [1,2]. Approximately 63% of all CRC occur in the colon, while 28% involve the rectum [3]. Surgery is the primary treatment for patients with non-metastatic CRC, whereas radiotherapy and chemotherapy are suitable strategies for metastatic CRC [4–6]. With progress in treatment, the mortality rate of CRC patients has significantly decreased [7,8]. Therefore, the development of novel therapeutic targets for CRC is necessary.

The zinc-finger structure, which is a tetrahedral structure for zinc (Zn^2+^) binding, is a common motif in nucleic acid-binding proteins [9]. Zinc-finger proteins usually function as transcription factors and regulate gene expression and disease progression [10,11]. Zinc-finger proteins also bind RNA and protein, and regulate the translation and posttranslational modification of coding genes and proteins, which are involved in disease progression [12,13]. Diverse zinc-finger proteins have been shown to be crucial regulators of cancer progression [14–16]. Promoter hypermethylation caused ZNF545 silencing in gastric cancer, and overexpression of ZNF545 efficiently inhibits cell proliferation and induces apoptosis [17]. ZBTB7A is mutated within the zinc finger domain in multiple types of human cancers, and these mutations lead to increased glycolysis and proliferation [18]. Elevation of Krüppel-associated box domain-containing zinc-finger family proteins by human endogenous retroviruses also leads to tumor suppression [19].

ZNF575 belongs to ZNF family, and its methylation status in newborns is influenced by antidepressant intake during pregnancy [20]. However, the function and underlying mechanism of ZNF575 in CRC is indistinct. In the present study, we aimed to investigate the function and mechanism of ZNF575 in CRC growth *in vitro* and in a mouse model. Furthermore, the expression ZNF575 were also determined in 150 pairs of CRC malignant tissues, followed with potential prognostic correlation analysis. The findings of our study would present evidence to clarify the role of ZNF575 in cancer.

## Materials and Methods

### Cell culture and treatment

All CRC cell lines and human normal epithelial cells (HCoEpiC) cells were purchased from the American Type Culture Collection (Manassas, VA, USA), and were tested via STR identification at West China Hospital in October 2021. Cells used in the present study were cultured in Dulbecco’s modified Eagle’s medium (DMEM; Thermo Fisher, MA, USA) supplemented with 10% fetal bovine serum (FBS; Thermo Fisher, MA, USA) and antibiotics (Thermo Fisher, MA, USA). Lentivirus-ZNF575 overexpression system and negative control lentivirus were purchased from Tsingke Bioscience Co., Ltd. (Chengdu, China) and used to infect HCT116 and RKO cells at a multiplicity of infection of 20. Puromycin (2 mg/ml) was added for selecting stable infected cells for further experiments.

### Cell proliferation assay

HCT116 (Control and ZNF575) and RKO (Control and ZNF575) cells were seeded into 96-well plates (1,000 cells/well). Cell Counting Kit 8 (10 μl, CCK-8; Dojindo, Kumamoto, Japan) was added to each well at 0, 24, 48, and 72 h after cell seeding and incubated for 2 h. The absorbance was measured at 450 nm using an MK3 microplate reader (Thermo Fisher, MA, USA).

### Colony formation assay

HCT116 (Control and ZNF575) and RKO (Control and ZNF575) cells were seeded into 6-well plates at a density of 2,000 cells/well. Ten days after cell seeding, the cells were incubated with 4% paraformaldehyde for 10 min at room temperature, then washed with PBS thrice and stained with crystal violet (Beyotime, Beijing, China) for 10 min at room temperature. The colonies in each well were counted and the number was analyzed.

### Flow cytometry

Annexin V-FITC/PI kit (KeyGEN Biotech, Nanjing, China) was used to detect apoptotic cells by flow cytometry. Briefly, HCT116-Control, HCT116-ZNF575, RKO-Control, and RKO-ZNF575 cells were collected, washed and incubated with cold PBS for 1 h, and resuspended in 500 μl binding buffer supplied by the kit. Next, the cells were incubated with 5 μl Annexin V-FITC for 15 min at room temperature, followed by incubation with 5 μl PI incubation for 5 min at room temperature. After washing with PBS for three times, the cells were loaded and analyzed for NoVo expression.

### Western blotting

HCT116 (Control and ZNF575) and RKO (Control and ZNF575) cells were collected and lysed with RIPA lysis buffer containing 1% protease inhibitor cocktail (Beyotime, Beijing, China) in ice for 15 min. After centrifugation for 15 min at 4°C (12,000 rpm), the protein in the supernatant was collected, and used for concentration determination by a BCA assay kit (Thermo Fisher Scientific, USA). For western blotting, total protein (10 μg) was separated using SDS-PAGE gel and transferred to PVDF membranes (Merck Millipore, MA, USA). Then, TBS/T buffer containing 5% non-fat milk was used for the membranes blocking for 1 h at room temperature. The membranes were incubated overnight with primary antibodies against p53, BAK, PUMA, and GAPDH (Cell Signaling Technology, MA, USA) at 4°C. After washing with TBS/T buffer for three times, the membranes were incubated with horseradish peroxidase (HRP)-conjugated secondary antibodies (ZB-2305 or ZB-2301) for 1 h at room temperature. The bands were detected using an iBrightTM CL1000 Instrument (Invitrogen, USA) with an ECL substrate (Merck Millipore, MA, USA).

### qPCR

HCT116 (Control and ZNF575) and RKO (Control and ZNF575) cells were collected for RNA extraction using the TRIzol reagent (Thermo Fisher Scientific, USA). The purity and concentration of the RNA were determined using NanoDrop 2000 (Thermo Fisher Scientific, USA). A total of 1 μg RNA was used for reverse-transcription using the PrimeScript™RT reagent Kit (Takara, Japan) following the manufacturer’s protocol. qPCR reaction was performed using SYBR Green Master Mix (Takara, Japan) with the following procedure: 95°C for 10 min, 45 cycles of 95°C for 5 s; 58°C for 30 s. 2^−ΔΔCt^ formula was used to calculate the relative expression.

### Animal study

BALB/c nude mice (female, 5–6 weeks old) were purchased from Gempharmatech (Chengdu, China) and maintained in SPF conditions. The collected HCT116 and RKO cells were washed five times with DMEM and resuspended in PBS before cell injection. After acclimation for one week, HCT116 and RKO cells were injected into the right flank of the mice. Tumor size was measured twice a week using a Vernier caliper and tumor volume was calculated after sacrificing mice. The animal study was approved by the Animal Care and Use Committee of Sichuan Provincial People’s Hospital, China.

### Immunohistochemical staining and TUNEL

Tumor slides and the CRC cohort were deparaffinized and rehydrated, followed by antigen retrieval for 3 min (high temperature and high pressure). The slides were then incubated with goat serum (Zsbio, Beijing, China; room temperature, 15 min) and washed with PBS three times. The slides were then incubated overnight with primary antibodies against ZNF575 (1:100, bs-13588R; Bioss, Shanghai, China) and p53 (1:500, 21891-1-AP; Proteintech, Wuhan, China) at 4°C. After incubation with secondary antibodies (Zsbio, Beijing, China; room temperature, 2 h), slides were stained with 3,3′-diaminobenzidine (Maixin, Fuzhou, China). Hematoxylin was used to stain cell nuclei. Apoptotic cells in tumor tissues were detected using the TUNEL assay (Beyotime, Beijing, China) following the manufacturer’s instructions. Cell nuclei were stained with DAPI (Beyotime, Beijing, China). All the slides were photographed using an Olympus BX600 microscope (Olympus, Tokyo, Japan).

### RNA sequencing

HCT116 cells (Control and ZNF575) were collected and used for RNA sequencing. RNA-Seq was performed by BaseBio (Chengdu, China). A fold change ≥1 and *p*-value < 0.05 was used as the screening criteria for differentially expressed genes.

### Chromatin immunoprecipitation (ChIP)

The SimpleChIP Plus Enzymatic Chromatin IP Kit (Cell Signaling Technology, CA, USA) was used for ChIP experiments, as indicated in a previous study [21]. Briefly, formaldehyde was added to the cell culture dishes for crosslinking the protein with DNA, and glycine was added to terminate the. Then the cells were collected, and the primary antibody against FLAG (20543-1-AP, Proteintech, Wuhan, China) was added and incubated overnight at 4°C. Then, after incubation with ChIP-Grade Protein G Magnetic Beads (Cell Signaling Technology, #9006) at 4°C for 2 h, the samples were gently swirled, mixed and incubated at 65°C for 30 min. Then NaCl and proteinase K were added for eluting chromatin at 65°C for 2 h. The protein was collected for western blotting, and DNA was collected for qPCR.

### Luciferase assay

P53 promoter-constructed luciferase reporter plasmid was purified. Lipofectamine 3000 Reagent (Thermo Fisher Scientific) was employed to transfect plasmids in 293T cells. After transfection with the p53 promoter-constructed luciferase reporter plasmid, 293T cells were seeded into 6-well plates (4 × 10^5^ cells per well) and transfected with the control and Flag-ZNF575 plasmids. Forty-eight hours later, the cells were collected and the protein were extracted. the Dual Luciferase Reporter Assay kit (GeneCopoeia, China) was employed to detect Firefly luciferase and Renilla luciferase detection following the manufacturer’s instructions.

### Colorectal cancer patient and prognosis analysis

In the present study, a cohort of 150 CRC malignant tissues and paired normal tissues was used for ZNF575 staining. The patients included in the cohort were confirmed to have CRC at Sichuan Provincial People’s Hospital. The prognosis data (overall survival and disease-free survival) of the patients were recorded following the approval of the Ethics Committee of Sichuan Provincial People’s Hospital. ZNF575 expression in the CRC cohort was determined by IHC staining. The expression of ZNF575 was scored by special pathologists using the following grading system (4, >40% positive; 3, 20%‒30% positive; 2, 10%‒20%; 1, 0%‒10% positive; 0, negative).

### Statistical analysis

GraphPad Prism 5 was employed to analyze the data in the present study. Data were expressed as mean ± standard deviation and analyzed by student’s *t*-test and chi-square test *(p*-value ≤ 0.05) (two-sided) was considered as a statistically significant difference. Kaplan–Meier method, followed by the log-rank test was used for prognosis analysis.

## Results

### Ectopic expression of ZNF575 inhibits CRC cell proliferation

**Figure 1:**
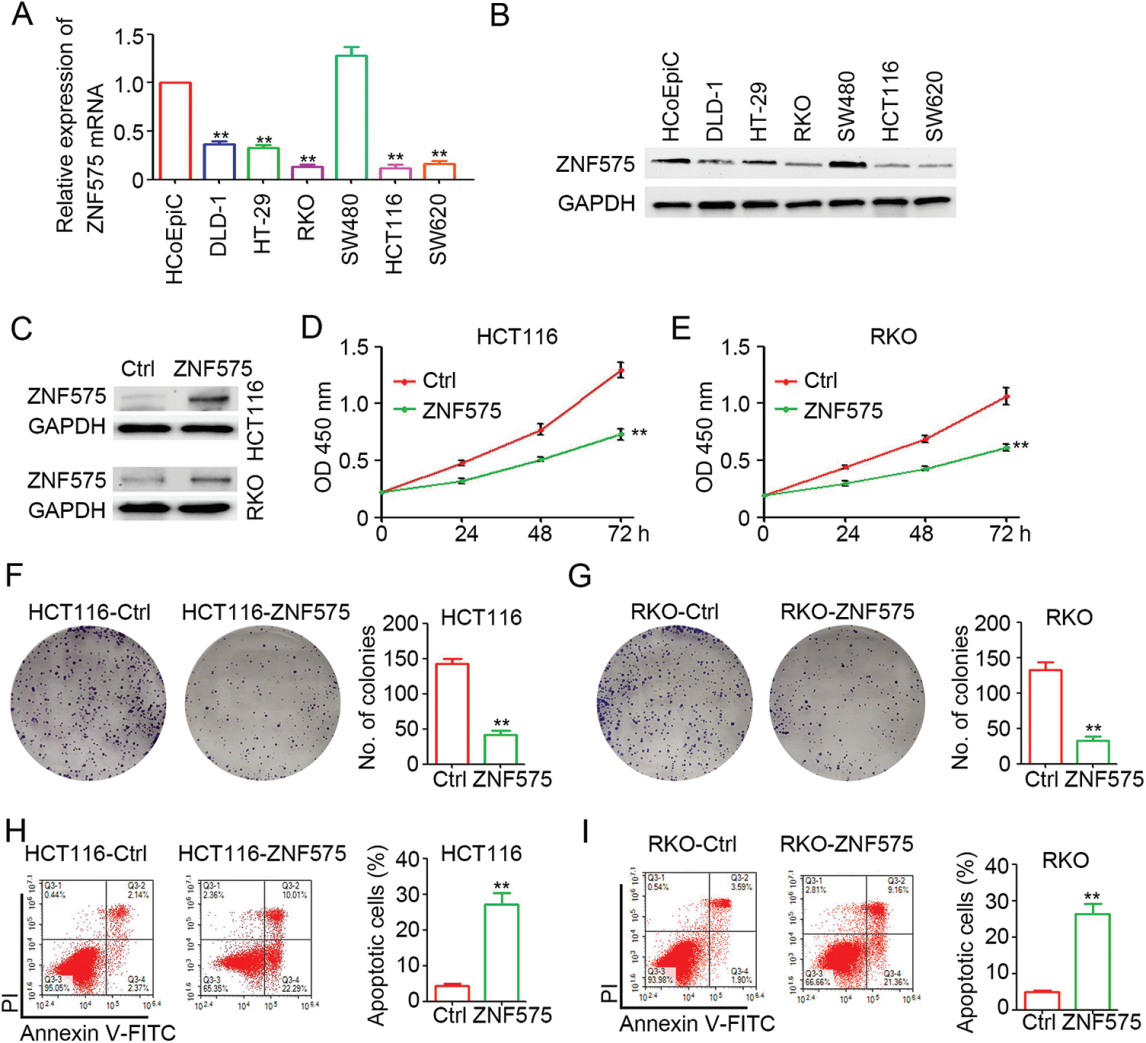
Ectopic expression of ZNF575 inhibits CRC cell proliferation. (A) Determination of ZNF575 mRNA expression in HCoEpiC and several CRC cell lines (DLD-1, HT-29, RKO, SW480, HCT116, SW620) by qPCR (n = 3, **p < 0.01, compared to HCoEpiC). (B) Determination of ZNF575 protein expression in HCoEpiC and several CRC cell lines (DLD-1, HT-29, RKO, SW480, HCT116, SW620) by qPCR. (C) Determination of ZNF575 protein expression in HCT116-Control (Ctrl), HCT116-ZNF575, RKO-Control and RKO-ZNF575 cells. (D and E) HCT116-Control, HCT116-ZNF575, RKO-Control and RKO-ZNF575 cells were seeded in 96-well plate (1,000 cells/well). The cell activity was measured by CCK-8 kit (n = 4, **p < 0.01). (F and G) HCT116-ControlControl, HCT116-ZNF575, RKO-ControlControl and RKO-ZNF575 cells were seeded in 6-well plate (2,000 cells/well). Ten days post cell seeding, the colony was stained by crystal violet. The number of colonies was counted and analyzed (n = 3, **p < 0.01). (H and I) HCT116-Control, HCT116-ZNF575, RKO-Control and RKO-ZNF575 cells were collected for apoptotic cells detection by flowy cytometry. The apoptotic percentage in each group was analyzed (n = 3, **p < 0.01).

To determine the expression of ZNF575, HCoEpiCs and several CRC cell lines were cultured for RNA and protein extraction. As shown in Fig. 1A, ZNF575 mRNA expression in DLD-1, HT-29, RKO, HCT116, and SW620 cells was significantly decreased compared with HCoEpiC cells, except in SW480 cells. Similar results were observed for ZNF575 protein expression in CRC cell lines (Fig. 1B). Lentivirus-based ZNF575 was used to infect HCT116 and RKO cells, which resulted in low ZNF575 expression. Significantly upregulated expression of ZNF575 was detected in HCT116-ZNF575 and RKO-ZNF575 cells compared to HCT116-Control and RKO-Control cells (Fig. 1C). The cell proliferation assay demonstrated that the ectopic expression of ZNF575 efficiently inhibited HCT116 and RKO cell proliferation (Figs. 1D and 1E). The colony formation assay also demonstrated fewer colonies in ZNF575-overexpressed HCT116 and RKO cells than in HCT116-Control and RKO-Control cells, respectively (Figs. 1F and 1G). Furthermore, more apoptotic cells were detected in HCT116-ZNF575 (Fig. 1H) and RKO-ZNF575 (Fig. 1I) cells than in HCT116-Control and RKO-Control cells, respectively. The above results indicated that ectopic expression of ZNF575 impaired CRC cell proliferation by promoting apoptosis.

### Ectopic expression of ZNF575 impairs CRC tumor growth in vivo

**Figure 2:**
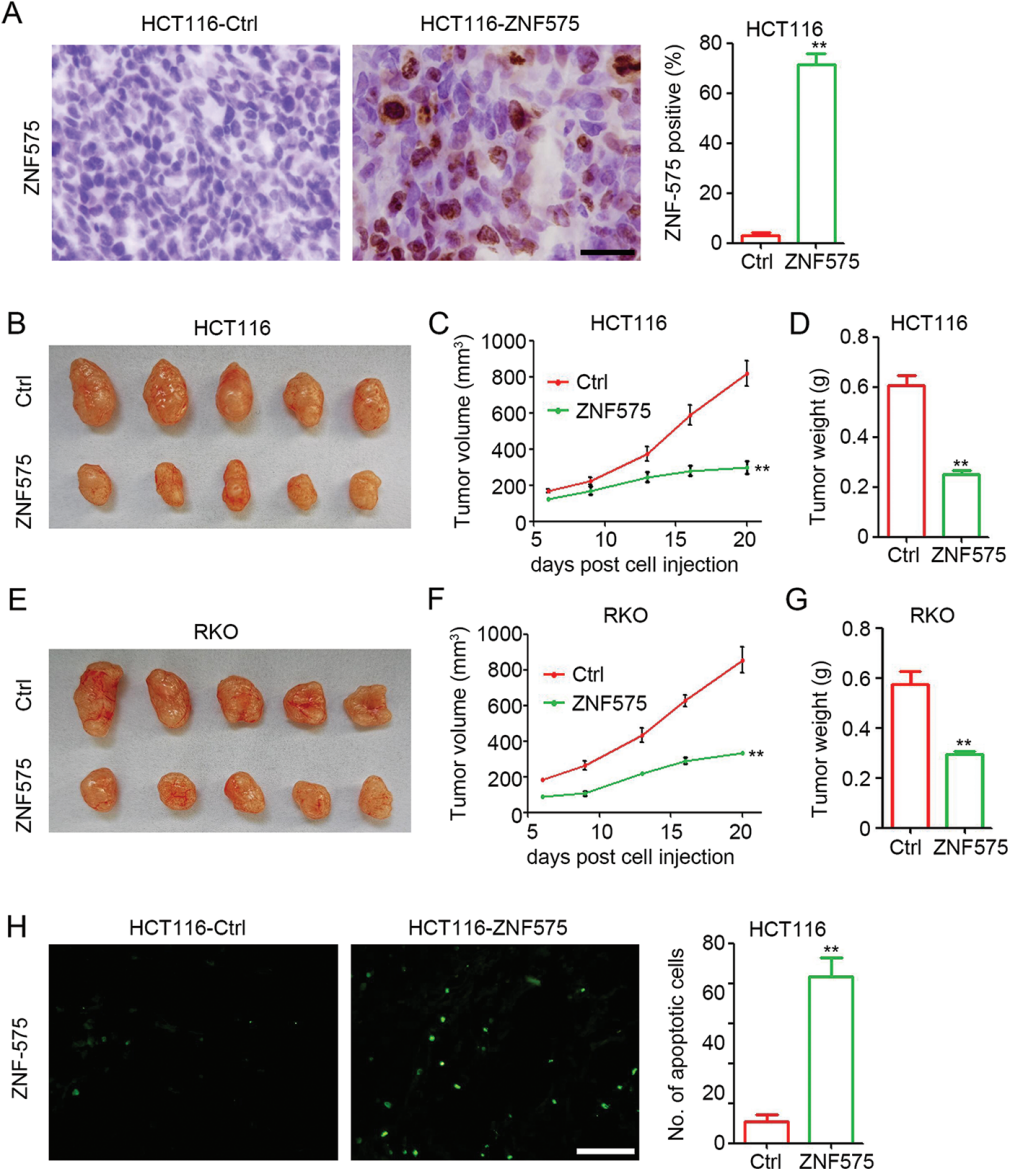
Ectopic expression of ZNF575 impairs CRC tumor growth in mice, (A) Detection of ZNF575 expression in HCT116-Control and HCT116-ZNF575 tumors by IHC staining (Scale bar = 100 μm). The percent of ZNF575 positive cells in HCT116-Control tumors and HCT116-ZNF575 tumors was analyzed (n = 3, **p < 0.01). (B) Image of HCT116-Control and HCT116-ZNF575 tumors at 20 days post cell injection. (C) Tumor growth curve of HCT116-Control and HCT116-ZNF575 tumors (n = 5, **p < 0.01). (D) Tumor weight of HCT116-Control and HCT116-ZNF575 tumors (n = 5, **p < 0.01). (E) Image of RKO-Control and RKO-ZNF575 tumors at 20 days post cell injection. (F) Tumor growth curve of RKO-Control and RKO-ZNF575 tumors (n = 5, **p < 0.01). (G) Tumor weight of RKO-Control and RKO-ZNF575 tumors (n = 5, **p < 0.01). (H) Detection of apoptotic cells in HCT116-Control and HCT116-ZNF575 tumors by TUNEL assay (Scale bar = 200 μm). The number of apoptotic cells in HCT116-Control tumors and HCT116-ZNF575 tumors was analyzed (n = 3, **p < 0.01).

**Figure 3:**
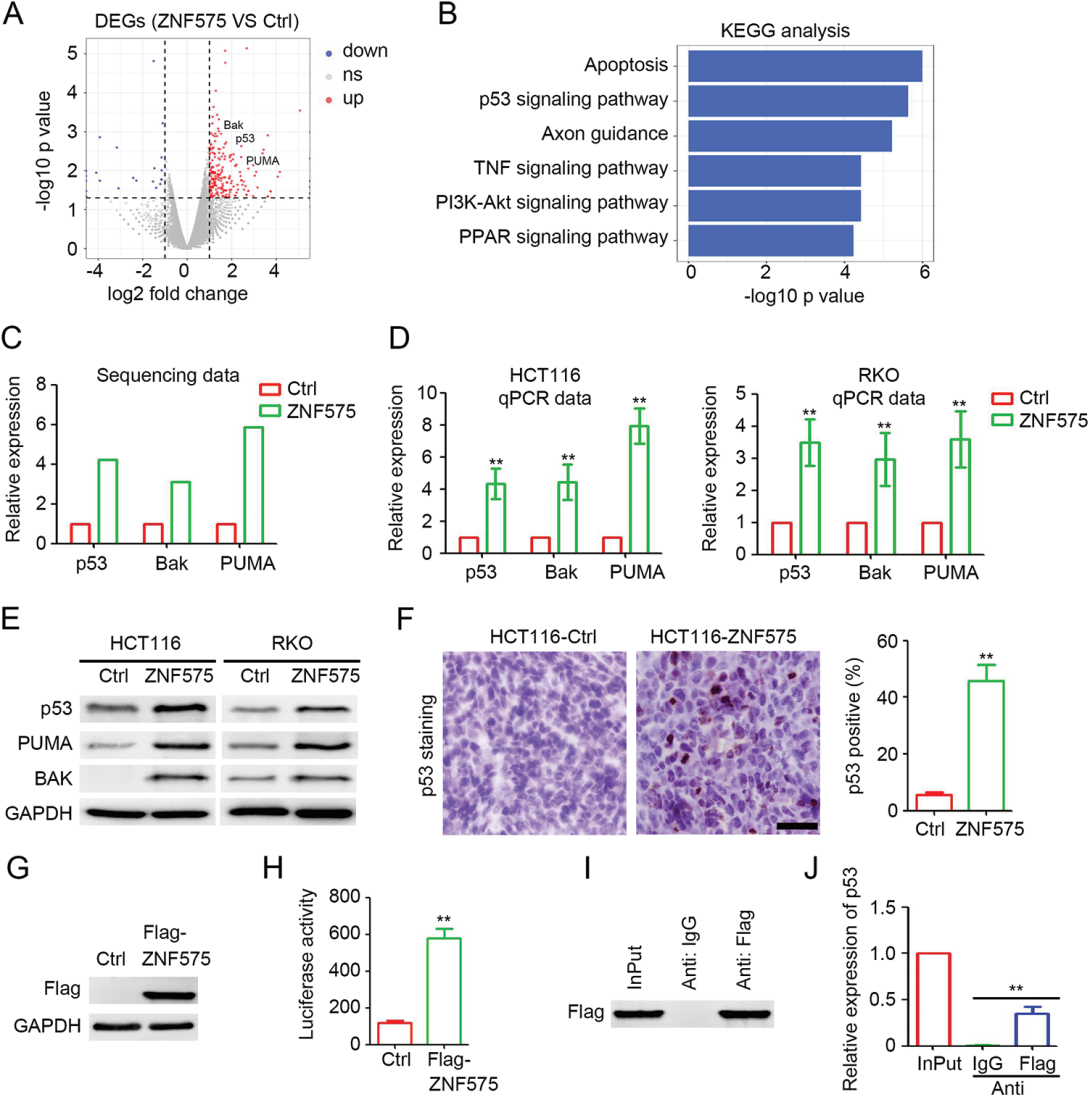
ZNF575 promotes p53 transcription and activates p53 signaling pathway. (A) Detection of deregulated protein coding genes in HCT116-ZNF575 cells, compared to HCT116-Control cells by RNA sequencing. (B) Function prediction of upregulated protein coding genes in HCT116-ZNF575 cells by KEGG analysis. (C) Expression of p53, Bak and PUMA in HCT116-Control and HCT116-ZNF575 cells which was determined by RNA sequencing. (D) Determination of p53, Bak and PUMA mRNA expression in HCT116-Control, HCT116-ZNF575, RKO-Control and RKO-ZNF575 cells by qPCR (n = 3, **p < 0.01). (E) Determination of p53, Bak and PUMA protein expression in HCT116-Control, HCT116-ZNF575, RKO-Control and RKO-ZNF575 cells by western blotting. (F) Detection of p53 expression in HCT116-Control and HCT116-ZNF575 tumors by IHC staining (Scale bar = 100 μm). The percent of p53 positive cells in HCT116-Control tumors and HCT116-ZNF575 tumors was analyzed (n = 3, **p < 0.01). (G) Detection of Flag expression in 293T cells transfected with control plasmid and Flag-ZNF575 overexpression plasmid. (H) The luciferase was detected by Dual-luciferase reporter assay (n = 3, **p < 0.01). (I) Western blotting analysis of Flag expression in 293T cells transfected with Flag-ZNF575 following with ChIP assay. (G) Detection of p53 promoter expression in Input, anti-IgG and anti-Flag group of ChIP assay (n = 3, **p < 0.01).

Next, to investigate the potential antitumor effect of ZNF575 in CRC, ZNF575-overexpressed HCT116 and RKO cells were used to establish xenograft tumors in mice. IHC results (Fig. 2A) confirmed the high expression of ZNF575 in HCT116-ZNF575 tumors, whereas no or low expression of ZNF575 was detected in HCT116-Control tumors. Our results indicated that ZNF575 significantly impairs HCT116 tumor growth with a 63.6% and 58.7% inhibition in tumor volume and tumor weight, respectively (Figs. 2B–2D, tumor volume, HCT116-Control 819.97 ± 153.82 mm^3^ *vs*. HCT116-ZNF575 297.41 ± 78.87 mm^3^; tumor weight, HCT116-Control 0.61 ± 0.09 g *vs*. HCT116-ZNF575 0.25 ± 0.04 g). ZNF575 also dramatically impaired RKO tumor growth with a 60.9% and 49.1% inhibition in tumor volume and tumor weight, respectively (Figs. 2E–2G, tumor volume, RKO-Control 854.82 ± 165.20 mm^3^ *vs*. RKO-ZNF575 334.43 ± 17.19 mm^3^; tumor weight, RKO-Control 0.58 ± 0.11 g *vs*. RKO-ZNF575 0.29 ± 0.03 g). The TUNEL assay demonstrated that ZNF575 induced more apoptotic cells in HCT116 tumors than in HCT116-Control tumors (Fig. 3H). Collectively, ZNF575 dramatically inhibited CRC tumor growth *in vivo*.

### ZNF575 promotes p53 transcription and activates p53 signaling pathway

To determine the downstream signaling pathways involved in ZNF575 regulation of CRC, HCT116-Control and HCT116-ZNF575 cells were used for RNA sequencing. As shown in Fig. 3A, 245 coding genes were upregulated in HCT116-ZNF575 cells, including p53, PUMA, and BAK, and the upregulated genes regulate apoptosis, p53 signaling pathway, and axon guidance (Fig. 3B). qPCR results in HCT116 and RKO cells confirmed the significant upregulation of p53, PUMA, and BAK in ZNF575 overexpressed cells, which is consistent with the results of RNA sequencing (Figs. 3C and 3D). Western blotting results also demonstrated that ZNF575 promoted p53, PUMA, and BAK expression in HCT116 cells (Fig. 3E). More p53-positive cells were detected in HCT116-ZNF575 tumors than in HCT116-Control tumors (Fig. 3F). Based on the significant upregulation of p53 mRNA and protein in ZNF575 overexpressed cells, we speculated that ZNF575 promotes p53 transcription. Therefore, a Flag-tagged ZNF575 overexpression plasmid was used to transfect 293T cells. Forty-eight hours later, the cells were collected and analyzed. Luciferase assay confirmed the overexpression of Flag (Fig. 3G) in Flag-ZNF575 transfected cells and the higher transcriptional activity of the p53 promoter (Fig. 3H). The ChIP assay indicated that Flag-ZNF575 directly interacted with the p53 promoter (Figs. 3I and 3J). These results suggested that ZNF575 promotes p53 transcription by directly interacting with the p53 promoter.

### P53 mediated ZNF575-caused inhibition of CRC proliferation

**Figure 4:**
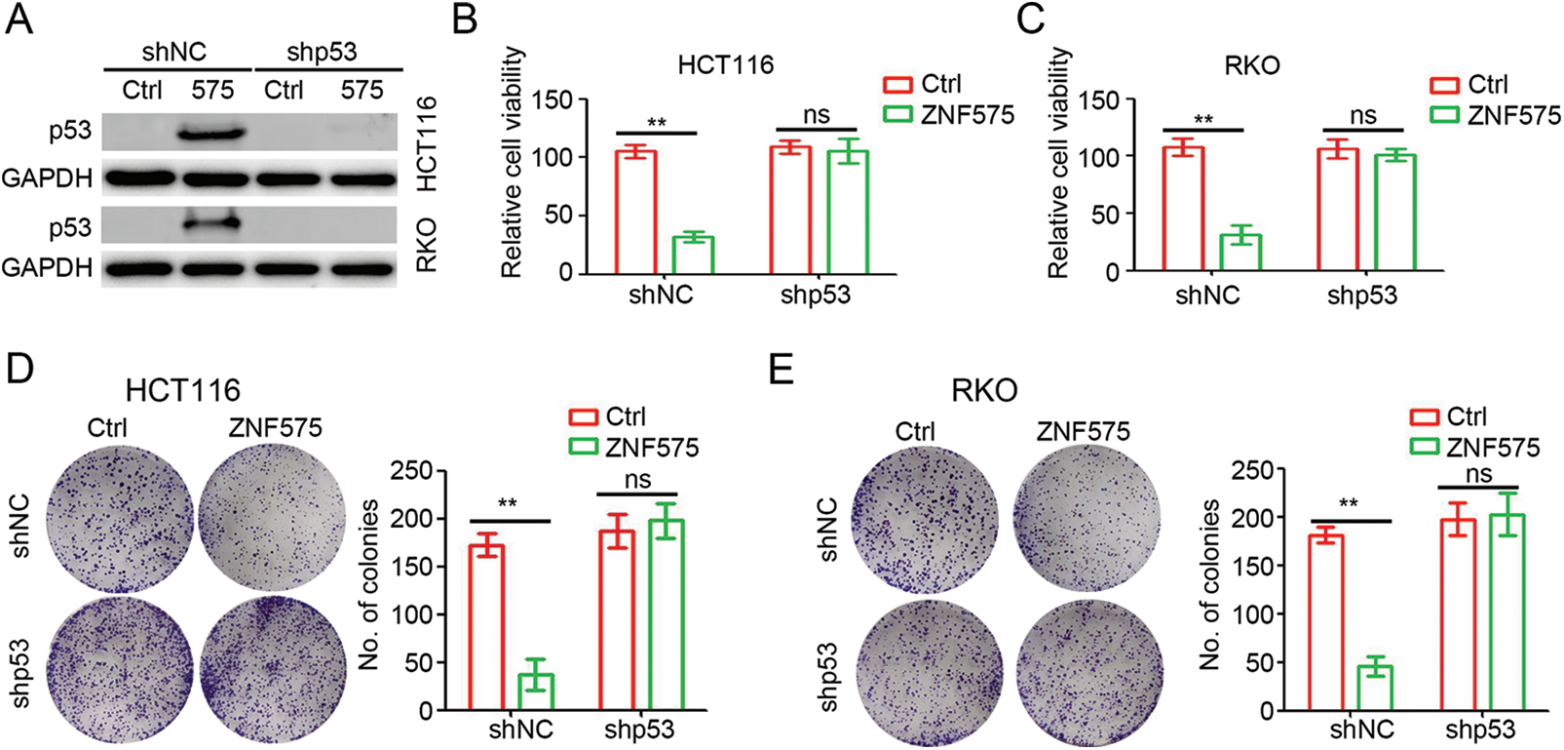
p53 plays a necessary role in ZNF575 inhibiting CRC proliferation. (A) Detection of p53 protein expression in HCT116-Control, HCT116-ZNF575, RKO-Control and RKO-ZNF575 cells that were transfected with shRNA and shp53 by Western blotting. (B and C) HCT116-Control, HCT116-ZNF575, RKO-Control and RKO-ZNF575 cells that were transfected with shRNA and shp53 were seeded in 96-well plate (1,000 cells/well). The cell activity was measured by CCK-8 kit at 72 h post cell seeding. The relative cell activity was analyzed (n = 4, **p < 0.01). (D and E) HCT116-Control, HCT116-ZNF575, RKO-Control and RKO-ZNF575 cells that were transfected with shRNA and shp53 were seeded in 6-well plate (2,000 cells/well). Ten days post cell seeding, the colony was stained and analyzed (n = 3, **p < 0.01; ns, no significant difference).

To determine the potential role of p53 in ZNF575 regulation of CRC, shRNA targeting p53 was employed to treat HCT116-ZNF575, HCT116-Control, RKO-ZNF575 and RKO-Control cells. Western blotting results suggested that no observed difference in p53 expression was detected between shp53 transfected cells (Fig. 4A). The cell proliferation assay demonstrated that ZNF575 significantly inhibited HCT116 and RKO cell proliferation, but inhibition of p53 expression blocked this inhibitory effect (Figs. 4B and 4C). Further colony formation assays also suggested that inhibition of p53 expression blocked the ZNF575-induced anti-tumor effect in CRCcells (Figs. 4D and 4E). Collectively, p53 plays a critical role during ZNF575 inhibition of CRC proliferation.

### ZNF575 correlates with prognosis in CRC patients

**Figure 5:**
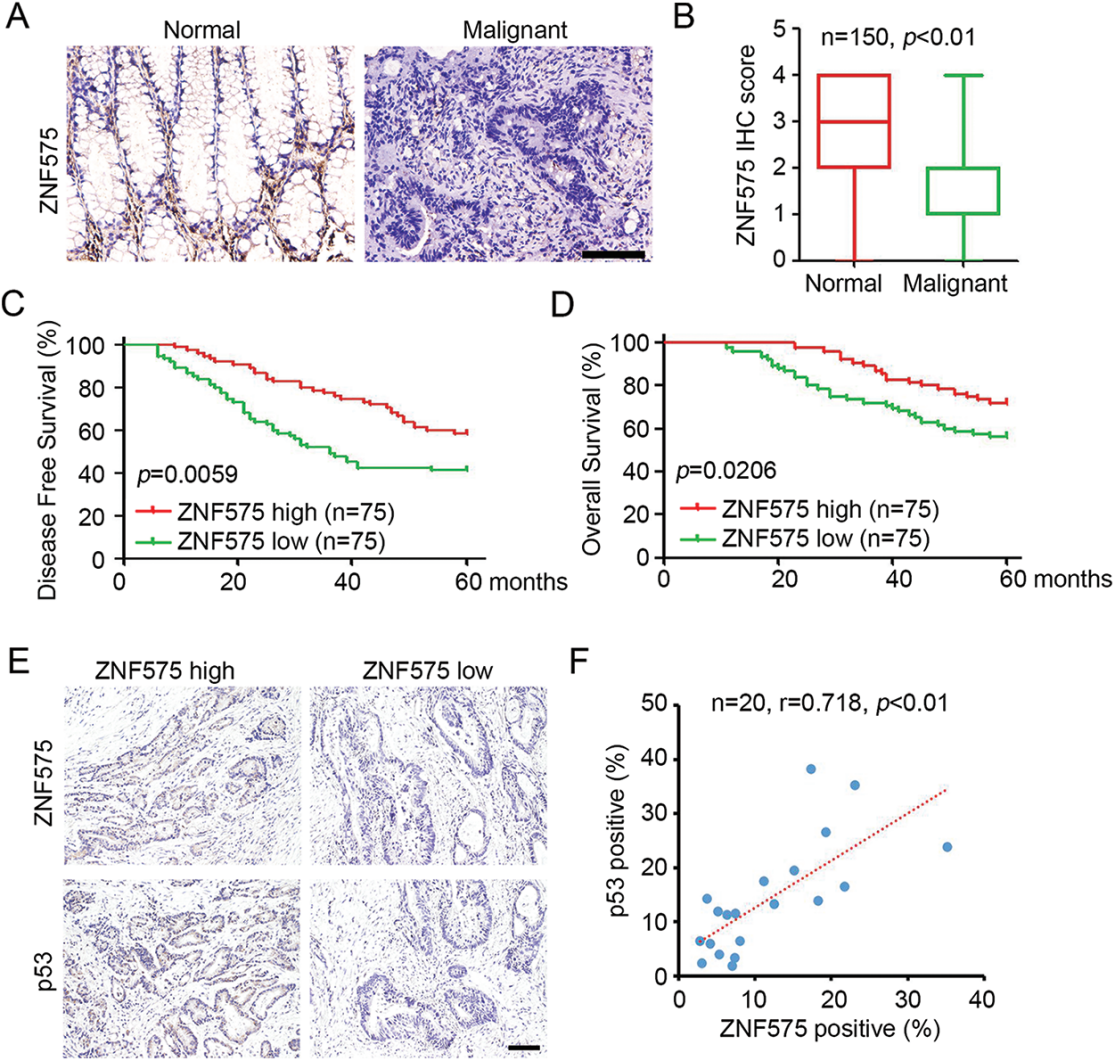
Downregulated ZNF575 correlates with positive prognosis of CRC patients. (A) Detection of ZNF575 expression in CRC malignant tissues and adjacent normal tissues of CRC cohort by IHC staining (Scale bar = 200 μm). (B) ZNF575 expression in each sample was scored as 0, 1, 2, 3 and 4. The score of ZNF575 in CRC malignant tissues and adjacent normal tissues was analyzed (n = 150, *p* < 0.01). (C and D) Based on the ZNF575 expression in each malignant tissues, CRC patients were divided into ZNF575-high and ZNF575-low group. Analysis of disease-free survival and overall survival percent in ZNF575-high and ZNF575-low group by Log-rank Test. (E) Detection of ZNF575 and p53 expression in 20 CRC malignant tissues by IHC staining (Scale bar = 50 μm). (F) Pearson correlation analysis of ZNF575 and p53 expression in 20 CRC malignant tissues.

The expression of ZNF575 in CRC patients was determined by IHC staining. The results demonstrated that positive ZNF575 cells were detected in CRC adjacent normal tissues, but no/low ZNF575 expression was detected in malignant CRC tissues (Fig. 5A). Further analysis based on IHC staining confirmed the dramatic downregulation of ZNF in malignant tissues compared to adjacent normal tissues. Patients were then divided into a ZNF575-high group and a ZNF575-low group based on IHC staining. Prognosis analysis demonstrated that patients in the ZNF575-high group got a higher disease-free survival rate (Fig. 5C) and overall survival rate (Fig. 5D) compared to those in the ZNF575-low expression group. Furthermore, the expression of ZNF575 and p53 in 20 CRC malignant tissues was determined. The results indicated that the tissues with high ZNF575 expression usually accompanied with high p53 expression (Fig. 5E). Pearson correlation analysis demonstrated that ZNF575 expression positively correlated with p53 expression in CRC malignant tissues (Fig. 5F). The above results demonstrated that ZNF575 correlated with a good prognosis of CRC patients.

## Discussion

**Figure 6:**
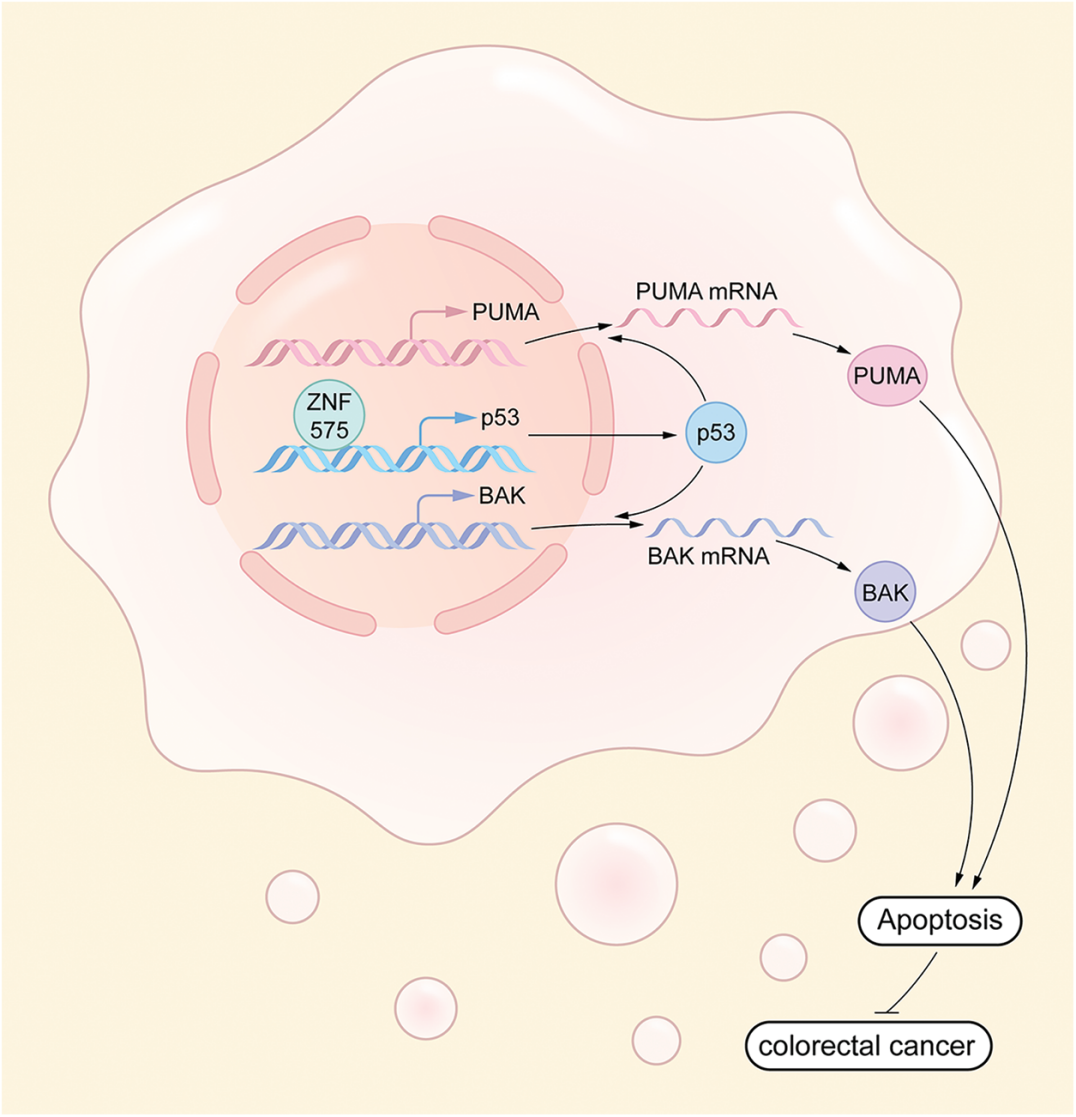
Molecular mechanism underlying ZNF575 inhibiting CRC growth ZNF575 promotes p53 expression and activates p53 signaling pathway via directly targeting p53 promoter, hence promotes BAK and PUMA transcription.

Our study is the first to clarify the function, underlying mechanism, and prognosis prediction role of ZNF575 in CRC. We demonstrated that the ZNF575 dramatically impaired CRC cell proliferation *in vitro*, accompanied by tumor growth inhibition in mice. The investigations indicated that ZNF575 promoted p53 expression and activated the p53 signaling pathway by directly targeting the p53 promoter (Fig. 6). Further results based on the CRC cohort suggested that ZNF575 was significantly downregulated in CRC tissues and positively correlated with the prognosis of CRC patients.

ZNF575 promotes p53 expression and activates p53 signaling pathway via directly targeting p53 promoter, hence promotes BAK and PUMA transcription.

Members of the zinc-finger protein family are deregulated in various cancers and function as potential predictors of prognosis in patients with cancer. Promoter CpG methylation status causes frequent downregulation of zinc finger and BTB/POZ domain-containing family protein 16 (ZBTB16) in breast cancer cell lines [22]. Pan-cancer analysis of 1552 patients indicated that zinc-finger antiviral protein (ZAP) is downregulated in liver, colon, and bladder cancer tissues, and correlated with poor survival of patients [23]. Furthermore, in a cohort of non-cancer controls and CRC patients, four zinc finger proteins (ZNF346, ZNF638, ZNF700, and ZNF768) were detected in CRC patients and adjacent normal tissues, and used as capture antigens for the detection of autoantibodies in CRC and conferred a specificity of 91.4% and sensitivity of 41.7% for the detection of cancer [24]. However, the expression of these finger proteins did not correlate with the prognosis of CRC patients [24]. Pan-cancer analysis based on the database of UALCAN indicated that ZNF-575 was downregulated in several cancer, including CRC, Uterine *Corpus* Endometrial Carcinoma (http://ualcan.path.uab.edu/index.html). I Our study demonstrated that ZNF575 was decrease in several CRC cell lines compared to that in normal human epithelial cells. Furthermore, IHC staining in the CRC cohort indicated that CRC malignant tissues had lower ZNF575 expression than the adjacent normal tissues. Prognosis analysis suggested that ZNF575 expression correlated with a higher 5-year disease survival and overall survival. These results clarify the role of ZNF575 in predicting the prognosis of CRC patients.

Zinc-finger proteins play different roles in various cancers. ZAP inhibits the aggressiveness of CRC cells and impairs APC deficiency-induced malignant colorectal cancer *in vivo* [23]. PRDI-BF1 and RIZ homology (PR) domain zinc finger protein 14 (PRDM14) function as oncogenes by promoting proliferation, sphere formation, distant metastasis, and chemotherapy resistance in breast and pancreatic cancer [25]. Silencing of PRDM14 impairs tumor growth and metastasis in breast and pancreatic cancers [25]. Myc-associated zinc-finger (MAZ) increases tumorigenesis of colitis-associated CRC and promotes the growth of human CRC cell lines [26]. Zinc-finger protein 277 (murine Zfp277) deficiency attenuates intestinal neoplasia and prolongs survival in APC^Min/+^ mice [27]. The novel findings in our study demonstrate the anti-tumor effect of ZNF575 in CRC, as evidenced by the inhibitory role of ZNF575 in CRC cell proliferation and tumor growth. Flow cytometry and TUNEL assay results confirmed the role of ZNF575 in promoting apoptosis in CRC cells. These results clarify the role of ZNF575 in cancer. But further experiments are needed to investigate the role of ZNF575 in regulating cancer cell metastasis, therapeutic responses, and the immune environment.

According to the suitable unit for DNA binding in the C2H2-type zinc-finger motif, most ZNF protein members are functioned as a transcription regulator for various genes, including Cyclin D2, VEGF, and CCND1 [28]. In our study, RNA sequencing suggested that ectopic expression of ZNF575 in CRC cells promoted the expression of p53 and its downstream targets BAK and PUMA. p53 is a well-known tumor suppressor that promotes the transcription of BAK, PUMA, p21, and other tumor suppressor genes [29]. A previous study also demonstrated that p53 transcription was repressed by ZNF322A, which represses by forming a complex with c-Jun [30]. Our results demonstrate that ZNF575 directly binds to the p53 promoter and induces p53 transcription. Inhibition of p53 expression could efficiently block ZNF575-induced inhibition of CRC cell proliferation. These results confirmed the direct binding between ZNF575 and the p53 promoter, and that p53-mediated ZNF575-caused inhibition of CRC cell growth. However, the binding site between ZNF575 and the p53 promoter should be investigated in future studies. Notably, p53 mutation is usually occurred in cancers and mutated p53 may play a diverse role in cancer proliferation. R248Q mutation in p53 actively functioned as an oncogene in several cancers through gain-of-function activities [31,32]. In the present study, we only indicated that e ZNF575 impaired cell proliferation in RKO and HCT116 cells with wild-type p53. Based on the promoting transcription activity of ZNF575 on p53 promoter, we speculated that ZNF575 would play a diverse role in CRC cells expression mutated p53 (R248Q/W), which also should be investigated in further study.

Collectively, we demonstrated that ZNF575 impairs CRC growth by directly targeting the p53 promoter and activating the p53 signaling pathway. Downregulation of ZNF575 is positively associated with prognosis of CRC patients. This study is the first to clarify the role of ZNF575 in CRC. Bu further experiments are needed to investigate the role and related molecular mechanism of ZNF575 in regulating other characteristics of cancer, such as metastasis, therapeutic responses, and immune environment.

## Data Availability

The data that support the findings of this study are available from the corresponding author, upon reasonable request.
